# Molecular determinants of epithelial mesenchymal transition in mouse placenta and trophoblast stem cell

**DOI:** 10.1038/s41598-023-37977-2

**Published:** 2023-07-06

**Authors:** Shipra Kanti Jena, Shreya Das, Shreeta Chakraborty, Rupasri Ain

**Affiliations:** 1 Division of Cell Biology and Physiology, CSIR-Indian Institute of Chemical Biology, 4, Raja S.C. Mullick Road, Jadavpur, Calcutta, West Bengal 700032 India; 2 Academy of Scientific and Innovative Research (AcSIR), Sector 19, Kamla Nehru Nagar, Ghaziabad, UP 201002 India

**Keywords:** Cell biology, Developmental biology

## Abstract

Trophectoderm cells of the blastocyst are the precursor of the placenta that is comprised of trophoblast, endothelial and smooth muscle cells. Since trophoectoderm cells are epithelial in nature, epithelial mesenchymal transition (EMT) of trophoblast stem (TS) cells might play pivotal role in placental morphogenesis. However, the molecular regulation of EMT during placental development and trophoblast differentiation still remained elusive. In this report, we sought to identify the molecular signature that regulates EMT during placental development and TS cell differentiation in mice. On E7.5 onwards the TS cells, located in the ectoplacental cone (EPC), rapidly divide and differentiate leading to formation of placenta proper. Using a real time PCR based array of functional EMT transcriptome with RNA from mouse implantation sites (IS) on E7.5 and E9.5, it was observed that there was an overall reduction of EMT gene expression in the IS as gestation progressed from E7.5 to E9.5 albeit the levels of EMT gene expression were substantial on both days. Further validation of array results using real time PCR and western blot analysis showed significant decrease in EMT-associated genes that included (a) transcription factors (Snai2, Zeb1, Stat3 and Foxc2), (b) extracellular matrix and cell adhesion related genes (Bmp1, Itga5, Vcan and Col3A1), (c) migration and motility- associated genes (Vim, Msn and FN1) and (d) differentiation and development related genes (Wnt5b, Jag1 and Cleaved Notch-1) on E9.5. To understand whether EMT is an ongoing process during placentation, the EMT-associated signatures genes, prevalent on E 7.5 and 9.5, were analysed on E12.5, E14.5 and E17.5 of mouse placenta. Interestingly, expression of these EMT-signature proteins were significantly higher at E12.5 though substantial expressions was observed in placenta with progression of gestation from mid- to late. To evaluate whether TS cells have the potential to undergo EMT ex vivo, TS cells were subjected to EMT induction, which was confirmed using morphological analysis and marker gene expression. Induction of EMT in TS cells showed similar gene expression profile of placental EMT. These results have broad biological implications, as inadequate mesenchymal transition leading to improper trophoblast-vasculogenic mimicry leads to placental pathophysiology and pregnancy failure.

## Introduction

Placenta, an ephemeral extra-embryonic organ, is crucial for the survival of mammalian embryo. During gestation it serves as a connecting link between developing fetus and mother. It has several vital functions, such as, facilitating nutrients supply to fetus, exchange of gases and waste products between mother and the fetus and is the source of pregnancy related hormones and growth factors, thereby supporting the immune system of fetus^1^. Various structural^2^, transcriptomic^3^ and epigenetic^4^ changes occur during placentation which are essential for proper feto-maternal development, defect in which lead to various pathological conditions^5^. Placenta comprised of different lineages of trophoblast cell, endothelial cell and smooth muscle cells, but it primarily originates from the outer epithelial trophectoderm cells of the blastocyst. On embryonic day 7.5, the ectoplacental cone (EPC) is evident at the implantation sites in mouse. EPC harbors the trophoblast stem (TS) cells and their niche. These TS cells give rise to trophoblast progenitor cells that rapidly divides and differentiate into various trophoblast lineages^6,7^. Interestingly, the trophoblast cells from the EPC also penetrates into the mesometrial decidua marking the first wave of trophoblast invasion during development in mouse. This first wave of invasion is essential in formation of definitive placenta. The second wave of trophoblast invasion leading to uterine spiral artery remodeling happens around mid-gestation in rodents^8^. Both waves of trophoblast invasion is absolutely essential in placental morphogenesis. It is likely that EMT is a prelude for these invasion processes. However, the genes involved in EMT during placental morphogenesis have not been illustrated comprehensively.

“Epithelial–mesenchymal transformation” was first described by Elizabeth Hay using a model of chick primitive streak formation^9^. Later, the term “transformation” was replaced with “transition”, owing to the reversibility of the process and for it is different than neoplastic transformation^10^. Discovery of the reverse process of EMT, called, mesenchymal–epithelial transition (MET) reflects the phenotypic plasticity of EMT^11,12^. The process of EMT involves acquisition of mesenchymal, fibroblast-like properties by epithelial cells. EMT is associated with a number of molecular and cellular events that include activation of transcription factors, reduction in expression of cell adhesion molecules, expression of specific cell-surface proteins, reorganization and expression of cytoskeletal proteins, production of extra-cellular matrix (ECM)-degrading enzymes^13,14^.

Interestingly, three classical types of EMT, i.e., (a) EMT associated with embryonic gastrulation^15^, (b) EMT in the context of inflammation and fibrosis^16,17^, (c) EMT associated with cancer^18–21^, do not describe EMT during placental morphogenesis. However, EMT in first trimester aborted placenta (ranging from 6 to 10 weeks gestational age) was demonstrated by 22. DaSilva-Arnold et al., 2015 ^22^. These authors compared EMT gene expression in extra-villous trophoblast (EVT) cells with cytotrophoblast (CTB) cells of the placenta and demonstrated up-regulation of mesenchymal markers and down regulation of epithelial markers in EVT compared to CTB. However, understanding EMT in placental morphogenesis throughout gestation cannot be done in humans due to ethical reasons. We, therefore, sought to analyze the genes that govern EMT during mouse placental morphogenesis. Our data highlight the important regulatory genes associated with EMT both in vivo in placenta and ex vivo in TS cells. These data will be useful in identifying factors involved in compromised placental development and placenta-associated developmental disorders.

## Results

### Functional EMT-transcriptome analysis highlights occurrence of EMT along with differentiation of multipotent TS cells on E7.5 and E9.5

**Figure 1:**
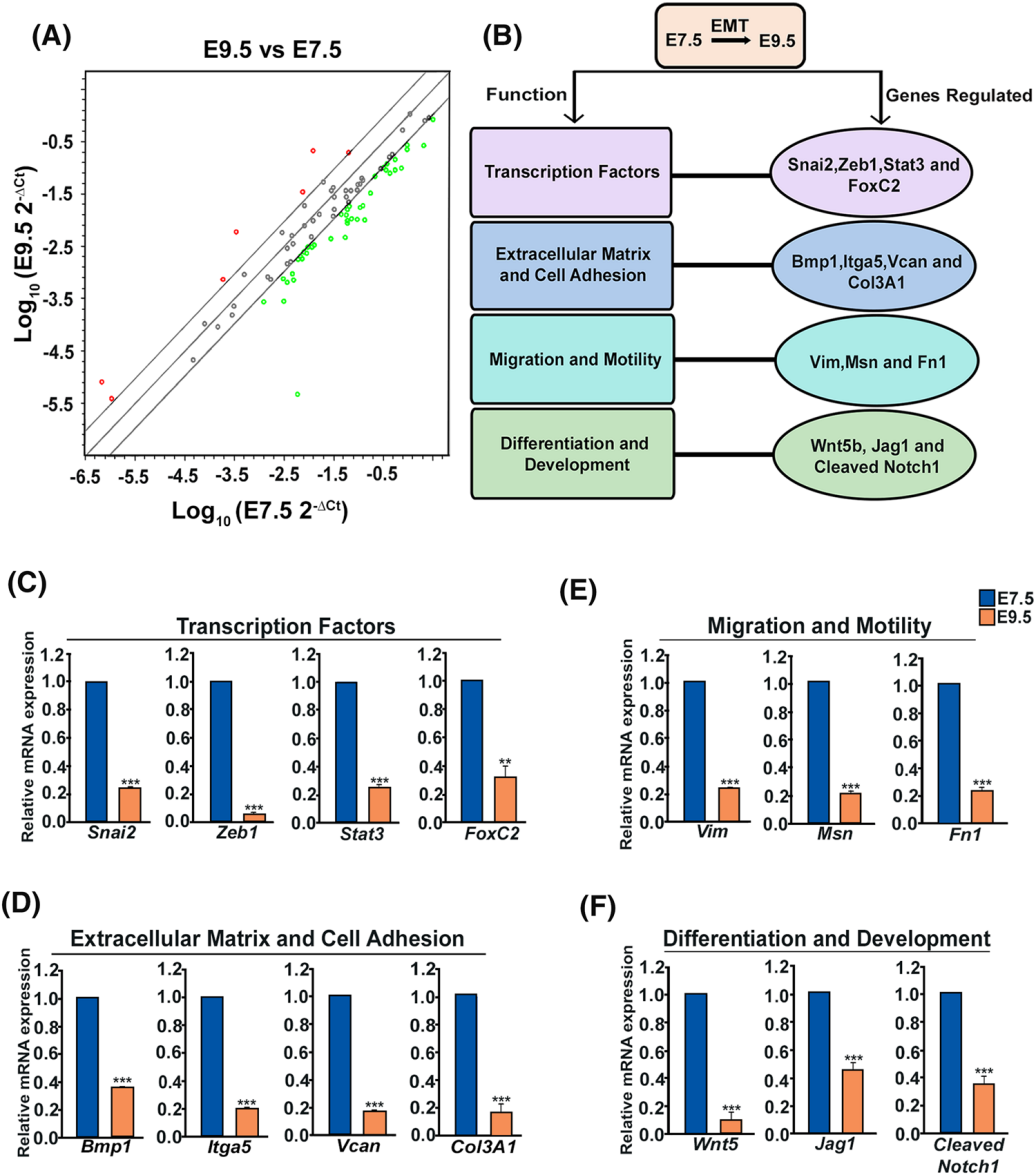
PCR array analysis profiling functional epithelial and mesenchymal transition (EMT) transcriptome and validation of EMT-associated transcripts in mouse implantation sites on E7.5 and E9.5. (**A**) Scatter plot of PCR array shows differential EMT gene expression of E7.5 and E9.5 mouse placental tissue. Genes represented by black dots (within black lines) are with similar expression patterns and close to the line of regression, whereas, up-regulated and down-regulated genes are represented by red dots (above black lines) and green dots (below black lines), respectively. The Scatter plot determines the log_10_ of normalized gene expression levels in E7.5 (x axis) versus that in E9.5 (y axis). (**B**) Functional annotation of 14 differentially regulated EMT genes in all three experiments at the IS on E7.5 and E9.5 are shown in a flowchart. Quantitative real-time PCR analysis shows significant decrease in mRNA level of EMT-associated genes at IS of E9.5 when compared with E7.5. Real time data are grouped according to functional annotation. (**C**) Transcription factors: *Snai2*, *Zeb1*, *Stat3*, *FoxC2.* (**D**) Extracellular matrix and Cell Adhesion genes: *Bmp1*,* Itga5*,* Vcan*,* Col3A1.* (**E**) Cell migration and motility genes: *Vim*, *Msn*, *Fn1.* (**F**) Genes associated with differentiation and development: *Wnt5B*,* Jag1*,* Notch1*. *Gapdh* was used as a reference gene for normalization. Error bars represents standard error of mean from three different biological replicates (n = 3, * p < 0.05, ** p < 0.01, and ***p < 0.005).

EPC containing trophoblast progenitor cells on E7.5 divides rapidly and differentiate along multi-lineage pathway leading to formation of a definitive placenta^23^. This is associated with invasion of the trophoblast cells into the decidua between E7.5 and E9.5^2^. Functional EMT-transcriptome was, therefore, analyzed using murine IS on E7.5 and E9.5, to understand whether TS cells undergo EMT during placental morphogenesis. To analyse the functional EMT-transcriptome, a real-time based RT^2^ Profiler™ PCR Array containing 84 EMT-associated genes was performed using RNA from IS on E7.5 and E9.5. Scatter Plot shows differential expression of EMT-related genes at the IS on E7.5 and E9.5 (Fig. 1A). Forty-one transcripts were found to be down-regulated at IS on E9.5 (Table S1). These transcripts met the recommended cut-off readings (C_t_ ≤ 30) in one of the two groups and showed fold change ≥ 2. Overall expression of EMT-associated transcripts decreased at the IS as gestation progressed from E7.5 to E9.5. Seven transcripts showing up-regulation in scatter plot were not analysed further as they did not accomplish the recommended cut-off in either the fold regulation or the C_t_ value in all three replicates. Differentially expressed EMT transcripts were functionally annotated into 4 major groups (Fig. 1B).

The differential expression of genes identified in the PCR-array was further validated by qRT-PCR using *Gapdh* as a reference gene. Real-time primers used for the qRT-PCR are listed in (Table 1). Out of 41 transcripts showing down-regulation in scatter plot, 14 were found to be differentially regulated at transcript levels as gestation day progressed from E7.5 to E9.5 in all three biological replicates (Fig. 1C–F; Table 2). These genes are broadly categorized into four groups. The first group of genes were comprised of transcription factors that included *Snai2* (*Slug*), *Zeb1*, *Stat3*, *FoxC2* (Fig. 1C). The second group was involved in formation of extracellular matrix and cell adhesion that included *Bmp1*, *Itga5*, *Vcan*, *Col3A1* (Fig. 1D). Similarly, genes related to cell migration and motility, which includes *Vim*, *Msn*, *Fn1* were significantly down regulated on E9.5 (Fig. 1E) along with the differentiation and development markers *Wnt5b*, *Jag1*, *Notch1* (Fig. 1F).

**Table 1:** List of primers used for real-time PCR analysis.

Sl. no.	Primers	GenBank accession number	Sequence (5′–3′)
1	*Snai2*	NM_011415.3	Fwd: AGAAGCCCAACTACAGCGAACT Rev: TGCCGACGATGTCCATACAG
2	*Zeb1*	NM_001360981.1	Fwd: GACTCCACGCCACCCAAA Rev: TCGTGAGGCCTCTTACCTGTGT
3	*Stat3*	NM_213659.3	Fwd: CTGGTGTCTCCACTTGTCTACCTCTAC Rev: GTGTCACACAGATGAACTTGGTCT
4	*FoxC2*	NM_013519.2	Fwd: ATCCGCCACAACCTGTCA Rev: GAAGCTGCCATTCTCGAACAT
5	*Bmp1*	NM_009755.3	Fwd: CCCTCCCAACAAAAACTGCAT Rev: AGCTTAGAGTCCGCCGTGAGT
6	*Itga5*	NM_001314041.1	Fwd: GCCGTACCCCAGACTTCTTTG Rev: GAGAGATGCGCTGGCAGATAT
7	*Vcan*	NM_001081249.1	Fwd: GAAACGGGAGATGGGCAT Rev: CCAGCGATGCTCATGTTTC
8	*Col3A1*	NM_009930.2	Fwd: AAATTCTGCCACCCCGAACT Rev: CAGTGCTTACGTGGGACAGT
9	*Vim*	NM_011701.4	Fwd: CCAGATTCAGGAACAGCATGTC Rev: TCAGCAAACTTGGACTTGTACCA
10	*Msn*	NM_010833.2	Fwd: CGGTCCTGTTGGCTTCTTATG Rev: CCACTGGTCCTTGTTGAGTTTG
11	*Fn1*	NM_010233.2	Fwd: AAGGTTCGGGAAGAGGTTGTG Rev GAGCTTAAAGCCAGCGTCAGA
12	*Wnt5b*	NM_009525.3	Fwd: CCAAGACGGGCATCAGAGA Rev: TGCATAGCTGAAGGCAGTCTCT
13	*Jag1*	NM_013822.5	Fwd: ATCCCGCACCCAGGATGT Rev: GGGCTGATGAGTCCCACAGT
14	*Notch 1*	NM_008714.3	Fwd: CATCCGTGGCTCCATTGTCTA Rev: TAAGGAATATTGAGGCTGCAAAGT
15	*GAPDH*	NM_001411845.1	Fwd: ATGTGTCCGTCGTGGATCTGA Rev: CTGTTGAAGTCGCAGGAGACAA

**Table 2:** List of validated genes from the array.

Sl. no.	Gene symbol	Accession no	Mean Ct E7.5	Mean Ct E9.5	Fold change	Gene description
1	Zeb1	NM_011546	21.9	34.32	− 1260.99	Zinc finger E-box binding homeobox 1
2	Vcan	NM_001081249	18.72	24.36	− 11.3703	Versican
3	Wnt5b	NM_009525	22.82	28.4	− 11.0047	Wingless-related MMTV integration site 5B
4	Col3a1	NM_009930	18.55	23.55	− 7.6942	Collagen, type III, alpha 1
5	Snai2	NM_011415	18.31	23.17	− 6.682	Snail homolog 2 (Drosophila)
6	Notch1	NM_008714	22.62	27.22	− 5.5737	Notch gene homolog 1 (Drosophila)
7	Vim	NM_011701	15.38	19.43	− 3.7944	Vimentin
8	Jag1	NM_013822	20.82	24.82	− 3.69	Jagged 1
9	Stat3	NM_011486	18.45	22.38	− 3.4751	Signal transducer and activator of transcription 3
10	Itga5	NM_010577	16.14	19.93	− 3.1648	Integrin alpha 5 (fibronectin receptor alpha)
11	Msn	NM_010833	16.72	20.5	− 3.1606	Moesin
12	Fn1	NM_ 010233	16.35	20.01	− 2.9009	Fibronectin 1
13	Bmp1	NM_009755	20.01	23.01	− 2.0256	Bone morphogenetic protein 1
14	Foxc2	NM_013519	26.17	28.72	− 2.07	Forkhead box C2

### EMT-signature proteins decreased at the implantation sites as gestation progressed from E7.5 to E9.5

**Figure 2:**
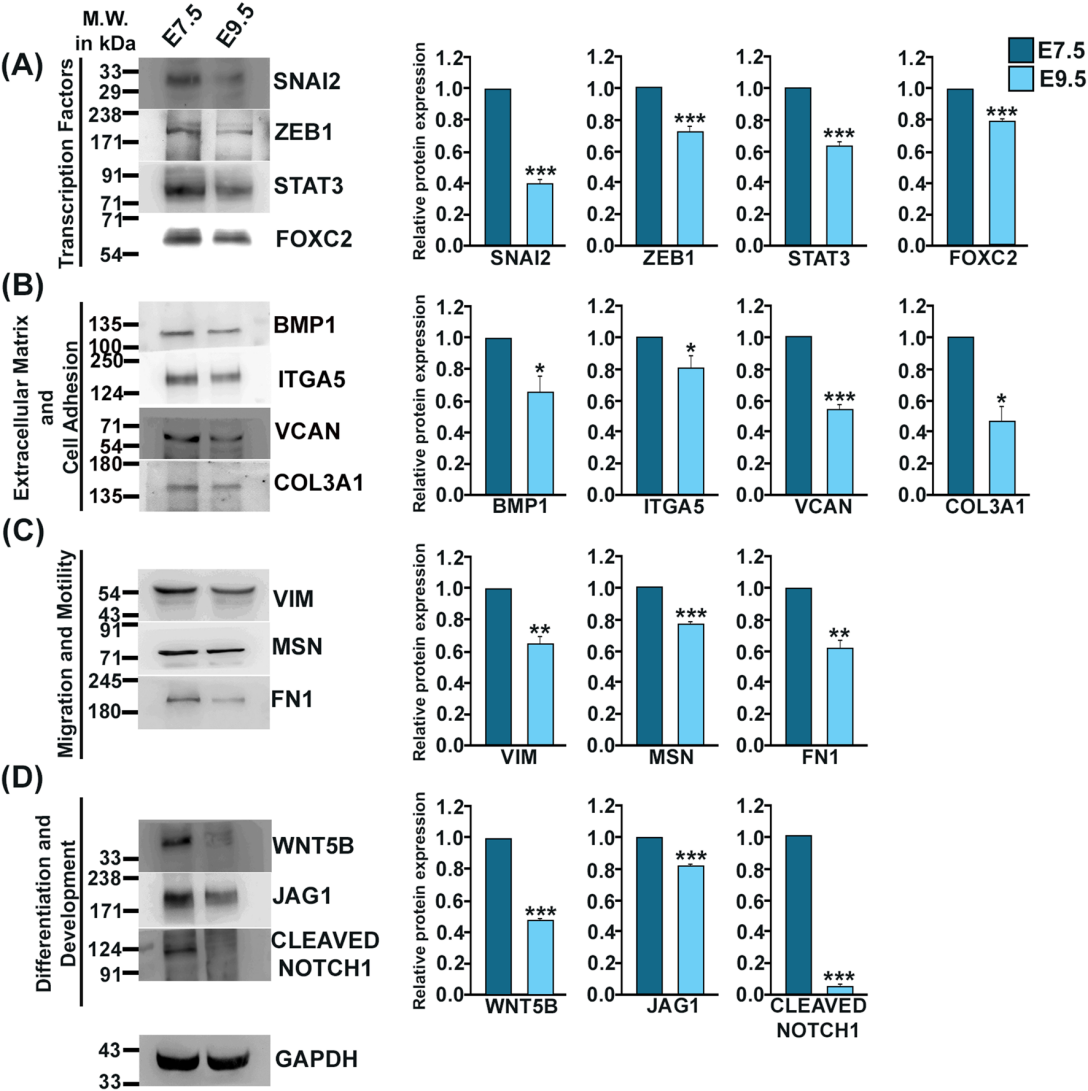
Decreased expression of EMT-associated proteins at implantation site on E9.5. Western Blot analysis of EMT-associated markers on E7.5–E9.5. Western Blots are grouped according to functional annotation. (**A**) Transcription factors: SNAI2, ZEB1, STAT3, FOXC2*.* (**B**) Extracellular matrix and Cell Adhesion markers BMP1, ITGA5, VCAN, COL3A1*.* (**C**) Proteins involved in cell migration and motility VIM, MSN, FN1*.* (**D**) Proteins associated with differentiation and development WNT5B, JAG1, NOTCH1. GAPDH was used as loading control and densitometry analysis was done using ImageJ software. Error bars represents standard error of mean from three different biological replicates (n = 3, *p < 0.05, **p < 0.01, and ***p < 0.005).

Since transcript levels do not always correlate with the corresponding protein abundance, for a better interpretation of our transcriptome analysis, we evaluated the protein levels of the identified EMT-associated transcripts by western blotting so as to conclude whether these markers are functionally significant or not (Fig. 2). On E9.5 EMT-related transcription factors, Snail homolog 2 (SNAI2), Zinc finger E-box binding homeobox 1(ZEB1), Signal transducer and activator of transcription 3 (STAT3), Fork head box C2 (FOXC2) significantly reduced in protein levels (Fig. 2A). The extracellular matrix and cell adhesion proteins, such as, Bone morphogenetic protein 1 (BMP1), Integrin alpha 5 (ITGA5), Versican (VCAN), Collagen, type III, alpha 1(COL3A1) were down-regulated with the progression of gestation day from E7.5 to E9.5 (Fig. 2B). Similarly, protein levels of cell migration and motility markers, such as, Vimentin (VIM), Moesin (MSN), Fibronectin (FN1) decreased significantly (Fig. 2C). Gradual decrease in protein expression of differentiation and development markers like Wingless-related MMTV integration site 5B (WNT5B), Jagged 1(JAG1), Notch gene homolog 1 (NOTCH 1) was observed as gestation progress from E7.5 to E9.5 (Fig. 2D). Original blots are shown in Fig. S1. A schematic representation of implantation site on E7.5 and utero-placental structure on E12.5 are depicted in Fig. 4A. Localization of VIMENTIN AND ZEB1 on E7.5 implantation sites are shown using immunohistochemical staining (Fig. 4B). Expression of VIMENTIN and ZEB1 was evident in EPC containing TS cells. This data indicates that EMT in trophoblast cells is a necessary prelude to the first wave of trophoblast invasion during formation of definitive placenta. Thus, with progression of gestation from E7.5 and E9.5 there was an overall reduction of EMT-related markers at the implantation sites.

### Gestation stage-dependent change of transcripts associated with EMT in mouse placenta

**Figure 3:**
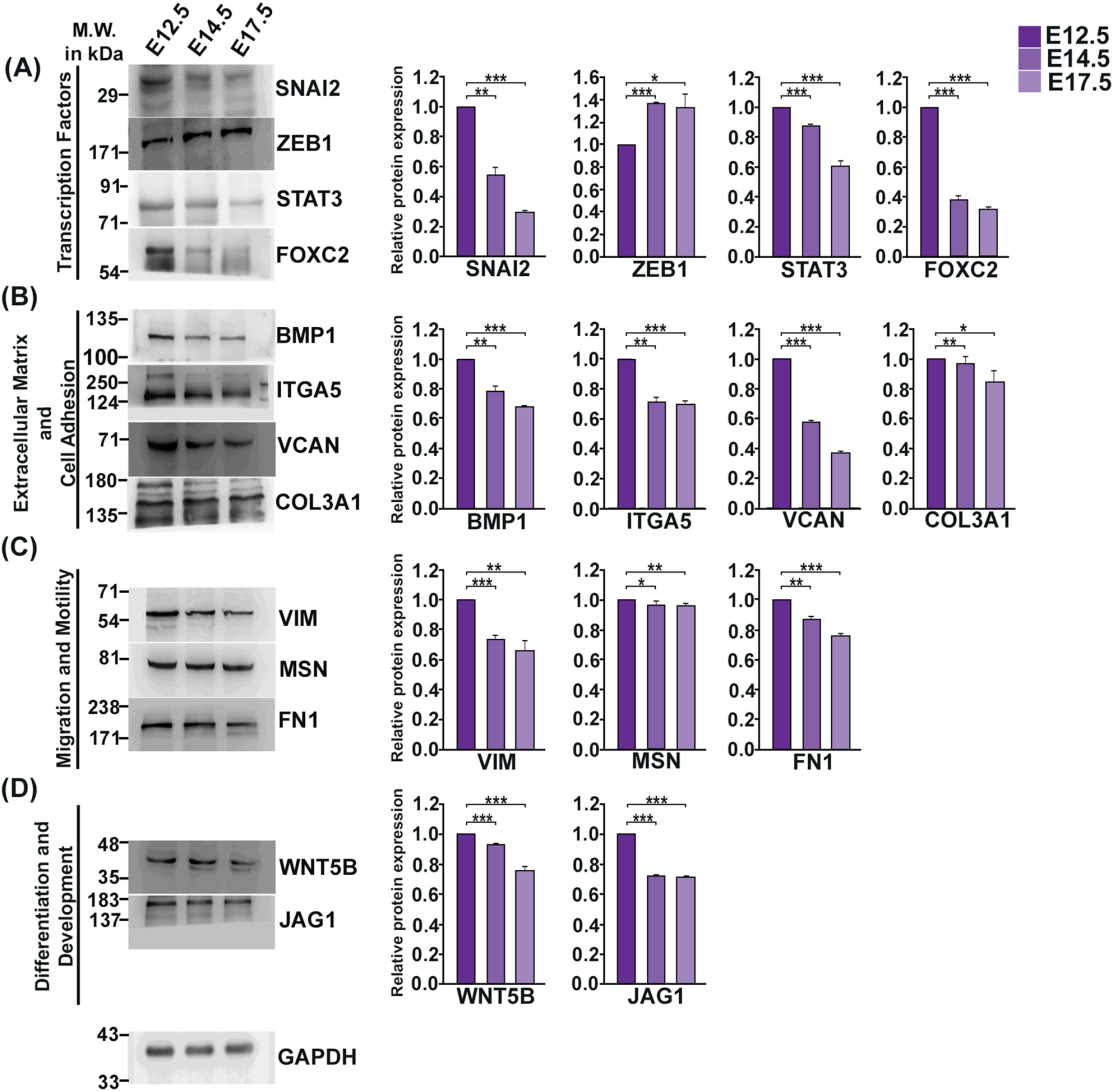
EMT-signature transcripts profiling in mouse placenta from mid to late gestation. Western Blot analysis of EMT-associated proteins in placenta from E12.5, E14.5 and E17.5. Western Blots are grouped according to functional annotation. (**A**) Transcription factors: SNAI2, STAT3, FOXC2. (**B**) Extracellular matrix and Cell Adhesion markers: BMP1, ITGA5, VCAN. (**C**) proteins involved in cell migration and motility: VIM, MSN, FN1*.* (**D**) Proteins associated with differentiation and development: WNT5B, JAG1. GAPDH was used as loading control for densitometric quantification using ImageJ software. Error bars represents standard error of mean from three different biological replicates (n = 3, *p < 0.05, **p < 0.01, and ***p < 0.005).

**Figure 4:**
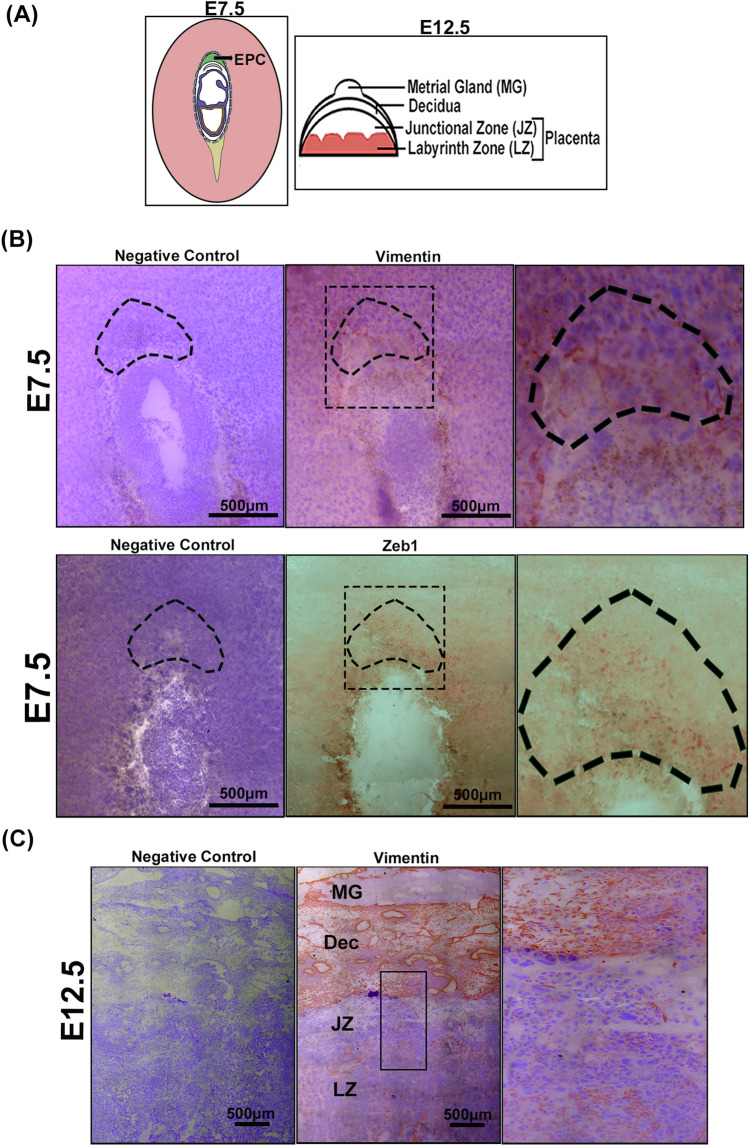
Cellular localization of EMT-markers in implantation site and placental tissue on E7.5 and E12.5, respectively. (**A**) Schematic representation of utero-placental sites on E7.5 and E12.5. (**B**) Immunohistochemical localization of VIMENTIN and ZEB1 (mesenchymal cell marker) on E7.5 are shown in the middle and right most panel. The leftmost panel shows the negative control. Area marked with dotted black line indicates the EPC region. Magnified image of the EPC region are shown (right-most). (**B**) Immunohistochemical staining of vimentin on E12.5 is shown in the middle panel and left most panel shows the negative control. Magnified image of the boxed area from the middle panel is shown on the right panel. Scale bar 500 µm. *MG* metrial gland, *Dec* decidua, *JZ* junctional zone of the placenta, *LZ* labyrinth zone of the placenta.

To understand whether EMT is an ongoing process during placental morphogenesis, EMT-associated markers identified on E7.5 and E9.5 were also analyzed in placenta with progression of gestation, i.e., from mid-gestation to late gestation using placental tissues from E12.5, E14.5 and E17.5. Expression of EMT-related markers, prevalent in E7.5 and 9.5, were tested in protein level using western blot analysis (Fig. 3). Interestingly, transcription factors associated with EMT, such as, SNAI2, STAT3, FOXC2 showed highest expression on E12.5, whereas, ZEB1 shows substantial presence throughout mid-late gestation (Fig. 3A). Extracellular matrix and cell adhesion markers, such as, BMP1, ITGA5, VCAN, COL3A1 gradually decreased with progression of gestation (Fig. 3B). Cell migration and motility markers, VIM and FN1 expression decreased gradually while there was negligible difference in expression of MSN as gestation progressed from E12.5 to E17.5 (Fig. 3C). Differentiation and development markers, such as, WNT5B declined gradually whereas JAG1 expression decreased from E12.5 to E14.5 and then remained unaltered till E17.5 (Fig. 3D). Full length blots are shown in Fig. S1. Furthermore, Immunohistochemical localization of vimentin (a well-known mesenchymal cell marker) on E12.5 murine placental tissue are shown in (Fig. 4C), which provides evidence for second wave of trophoblast invasion where cells from junctional zone migrates to decidua for uterine spiral artery remodelling. Taken together, it was observed that as gestation progressed from mid- to late (E12.5–E17.5) there was gradual decrease in expression of EMT-associated markers. Highest expression of EMT-signature proteins was found in placenta from E12.5 and adequate expression was observed on E14.5 and E17.5.

### TS cells possess the potential to undergo EMT ex vivo

**Figure 5:**
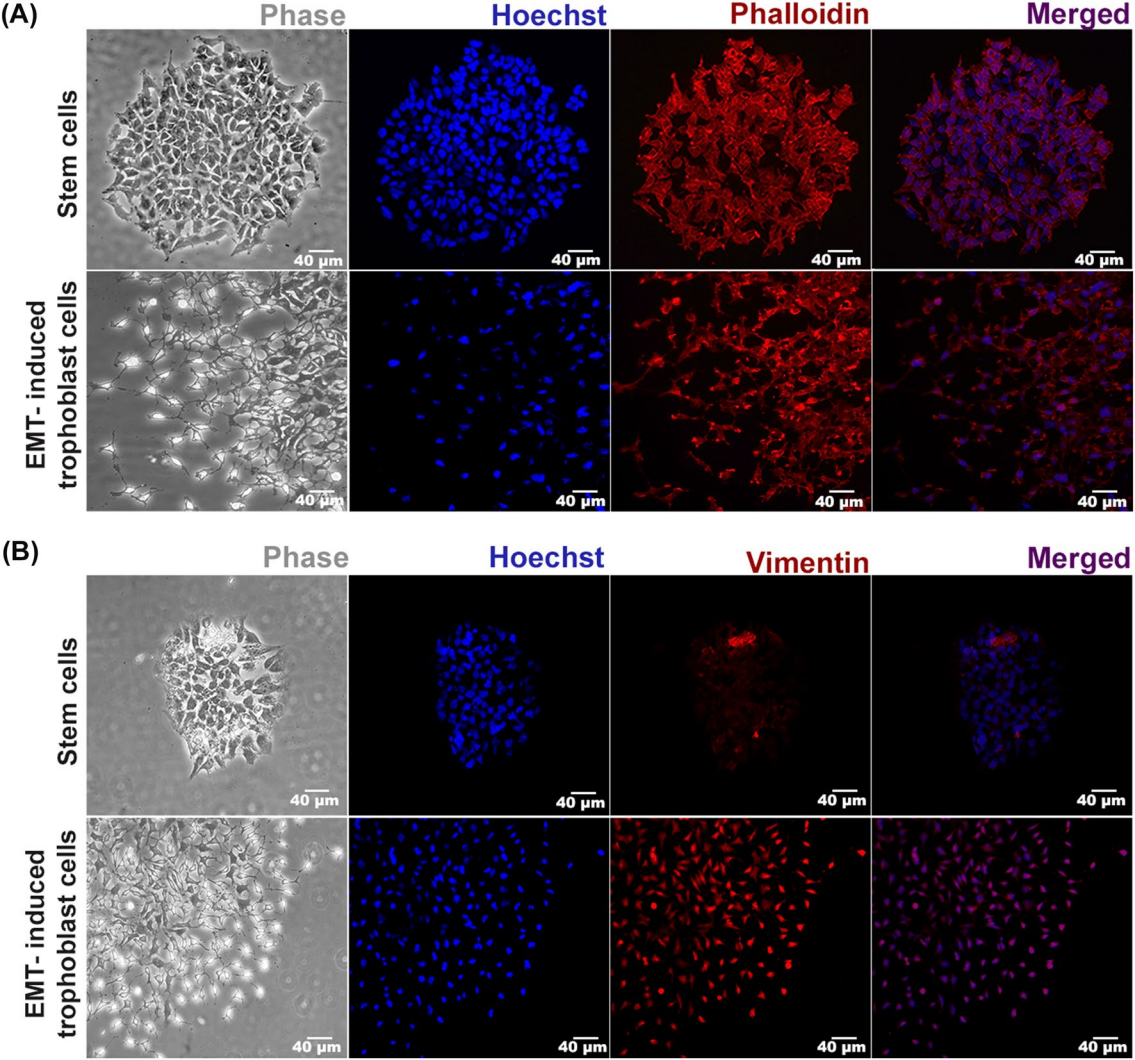
Phenotypic characterization of TS cells upon EMT induction. (**A**) Morphological characterization of TS cells and EMT-induced trophoblast cells were done using Hoechst–Phalloidin staining. Photo-micrographic images on day 5 shows TS cells cytoskeleton resembles colony like structure while upon EMT-induction migratory cytoskeleton are visible. Leftmost (first) panel: phase contrast, second panel: nuclear staining with Hoechst (blue), third panel: cytosol staining of actin filaments using DyLight 554 Phalloidin (red) and rightmost (fourth) panel: merged. Scale bar 40 µm. (**B**) Immunofluorescence staining of trophoblast cells using anti-VIM antibody (red). Nuclei were counter-stained with Hoechst (blue). Scale bar 40 µm.

Establishment of mouse TS cell line by Prof. Rossant’s group paved the way in understanding molecular regulation of mouse placental development ex vivo. These cells can be maintained in proliferative state and can also be differentiated. We used these TS cells to verify whether they are capable of undergoing EMT. TS cells were treated with EMT-inducer cocktail and morphology of the cells were analyzed by phase contrast microscopy and by fluorescence staining of the nuclei and the cytoskeleton using Hoechst-Phalloidin. Upon induction of EMT, the cells started to migrate out of their colony like structures as observed by phase contrast microscopy. Migratory cytoskeletons were visible in EMT-induced trophoblast cells in contrast to TS cells in Hoechst-Phalloidin-stained cells (Fig. 5A). EMT induction in TS cells was further verified by immunofluorescence staining using VIM antibody. VIM-positive cells increased remarkably upon EMT induction (Fig. 5B).

**Figure 6:**
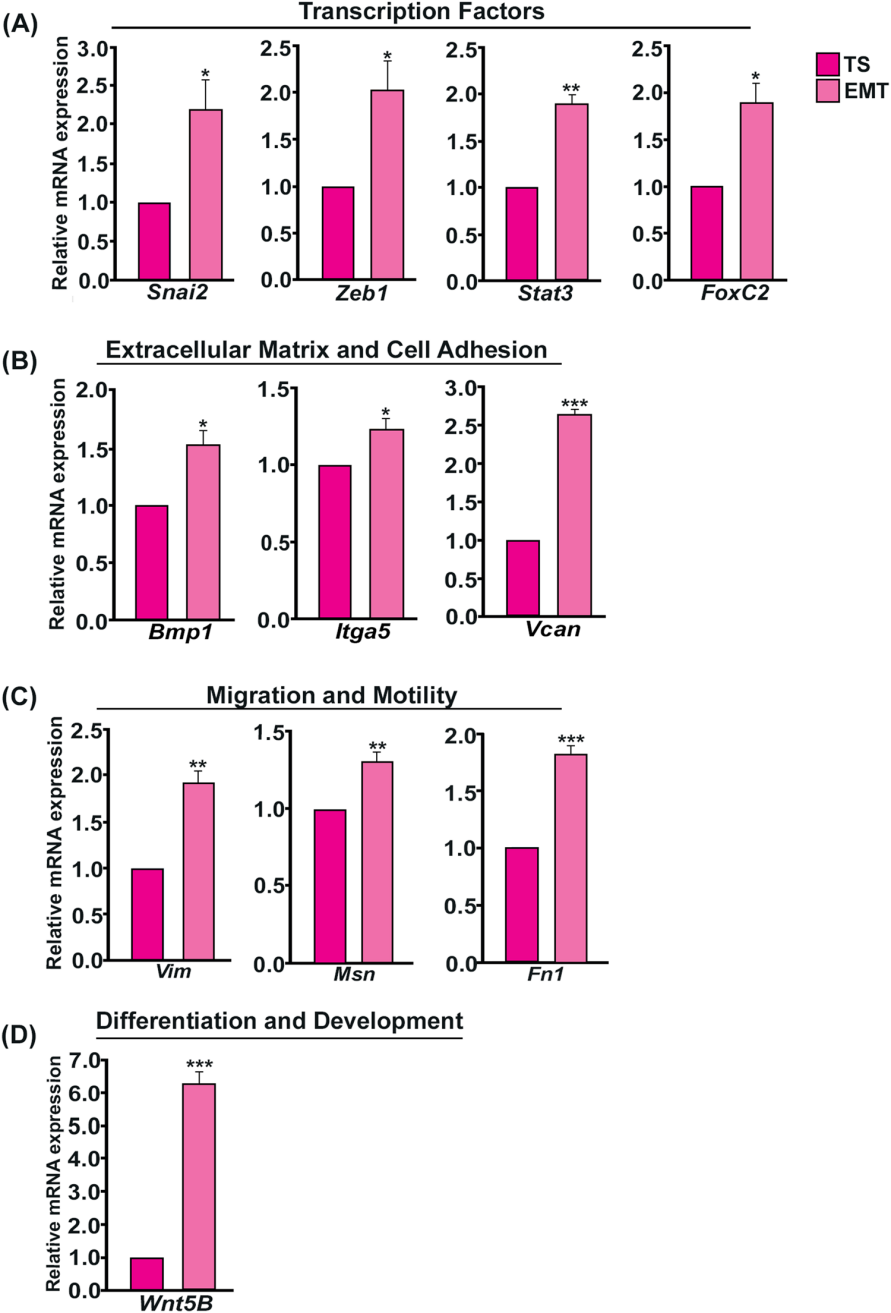
Real-time PCR analysis of EMT-signature genes in TS cells and EMT-induced trophoblast cells. Quantitative real-time PCR analysis shows significant increase in mRNA level of EMT-associated genes upon EMT induction in TS cells. Real time data are grouped according to functional annotation. (**A**)Transcription factors: *Snai2*, *Zeb1*, *Stat3*, *FoxC2.* (**B**) Extracellular matrix and Cell Adhesion genes *Bmp1*, *Itga5*, *Vcan.* (**C**) Cell migration and motility genes *Vim*, *Msn*, *Fn1.* (**D**) Genes associated with differentiation and development *Wnt5B*. *Gapdh* was used as a reference gene for normalization of specific mRNA. Error bars represents standard error of mean from three different biological replicates (n = 3, *p < 0.05, **p < 0.01, and ***p < 0.005).

Following phenotypic characterization of EMT induction using microscopy, genotypic confirmation of EMT induction was carried out by transcript and protein analysis. Analysis of EMT-signature transcripts identified in mouse placenta in TS cells and EMT-induced trophoblast cells revealed significant increase in EMT-associated transcription factors *Snai2* (*Slug*), *Zeb1*, *Stat3 and FoxC2* (Fig. 6A). Increased transcript levels of extracellular matrix and cell adhesion molecules, such as, *Bmp1*, *Itga5*, *Vcan* were also observed in EMT-induced trophoblast cells (Fig. 6B). Similarly, in EMT-induced trophoblast cells, mRNAs related to cell migration and motility which includes *Vim*, *Msn*, *Fn1* were significantly up-regulated (Fig. 6C) along with the differentiation and development markers *Wnt5b* (Fig. 6D).

**Figure 7:**
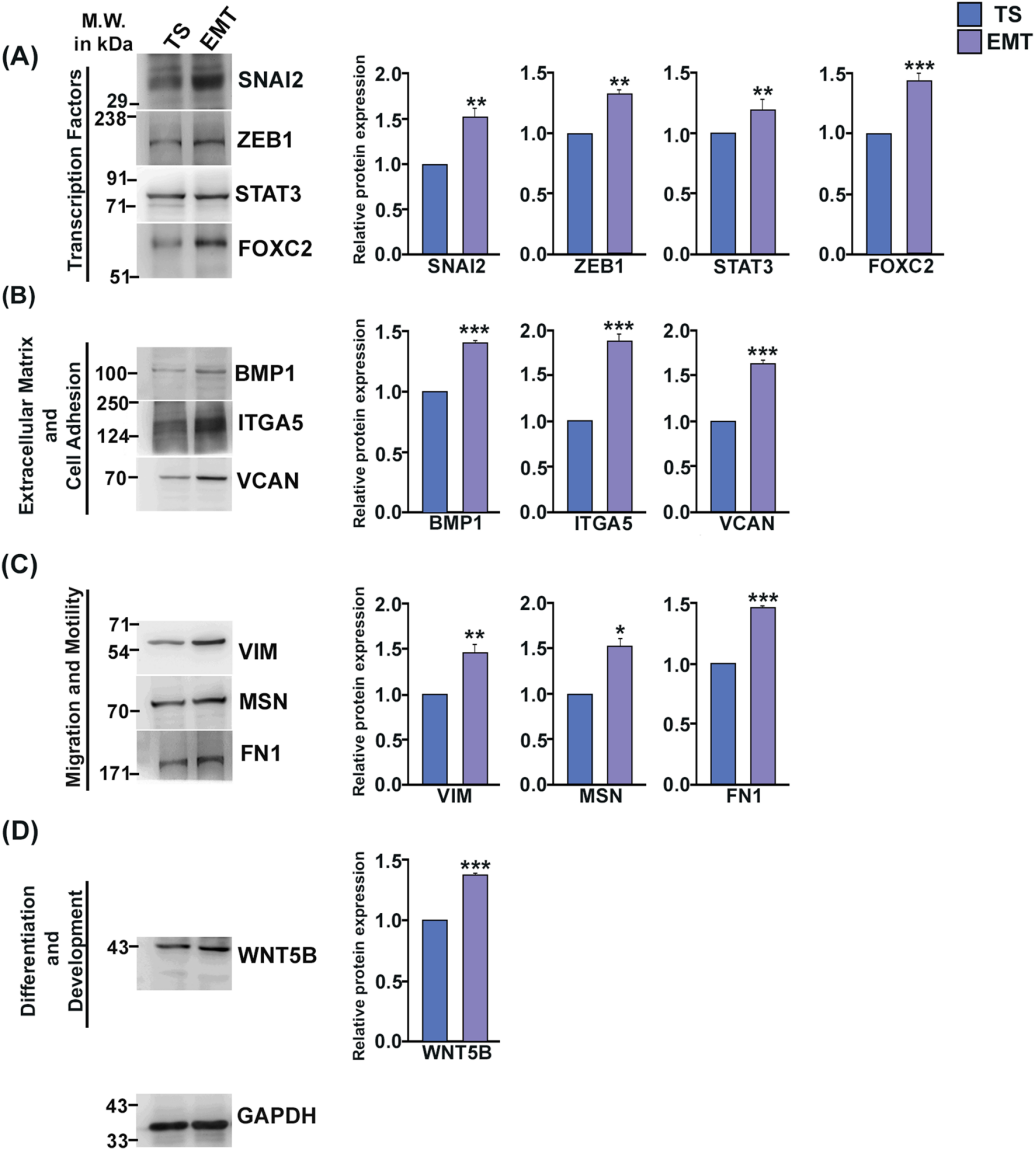
EMT-induction in TS cells is associated with increased expression of EMT-associated proteins. Western Blot analysis of EMT-associated markers in TS cells and EMT-induced trophoblast cells. Western Blots are grouped according to functional annotation. (**A**)Transcription factors: SNAI2, ZEB1, STAT3, FOXC2*.* (**B**) Extracellular matrix and Cell Adhesion markers BMP1, ITGA5, VCAN*.* (**C**) proteins involved in cell migration and motility VIM, MSN, FN1*.* (**D**) Proteins associated with differentiation and development WNT5B. GAPDH was used as loading control for normalization and densitometry analysis was done using ImageJ software. Error bars represents standard error of mean from three different biological replicates (n = 3, *p < 0.05, **p < 0.01, and ***p < 0.005).

Further validation of EMT induction in TS cells was done by evaluating protein levels of EMT-signature markers using western blot analysis. Transcription factors associated with EMT, such as, SNAI2, STAT3, FOXC2 showed enhanced expression in EMT-induced cells (Fig. 7A). Upon EMT-induction there was significant increase in the expression of extracellular matrix and cell adhesion markers, BMP1, ITGA5, VCAN (Fig. 7B) and Cell migration and motility markers VIM, FN1 and MSN (Fig. 7C). Differentiation and development markers, such as, WNT5B also showed increased expression in EMT-induced trophoblast cells (Fig. 7D). Full length blots are shown in Fig. S1. Thus, EMT appears to be a hallmark event in placental morphogenesis throughout gestation, which is verified further ex vivo using TS cell model of placental development.

**Figure 8:**
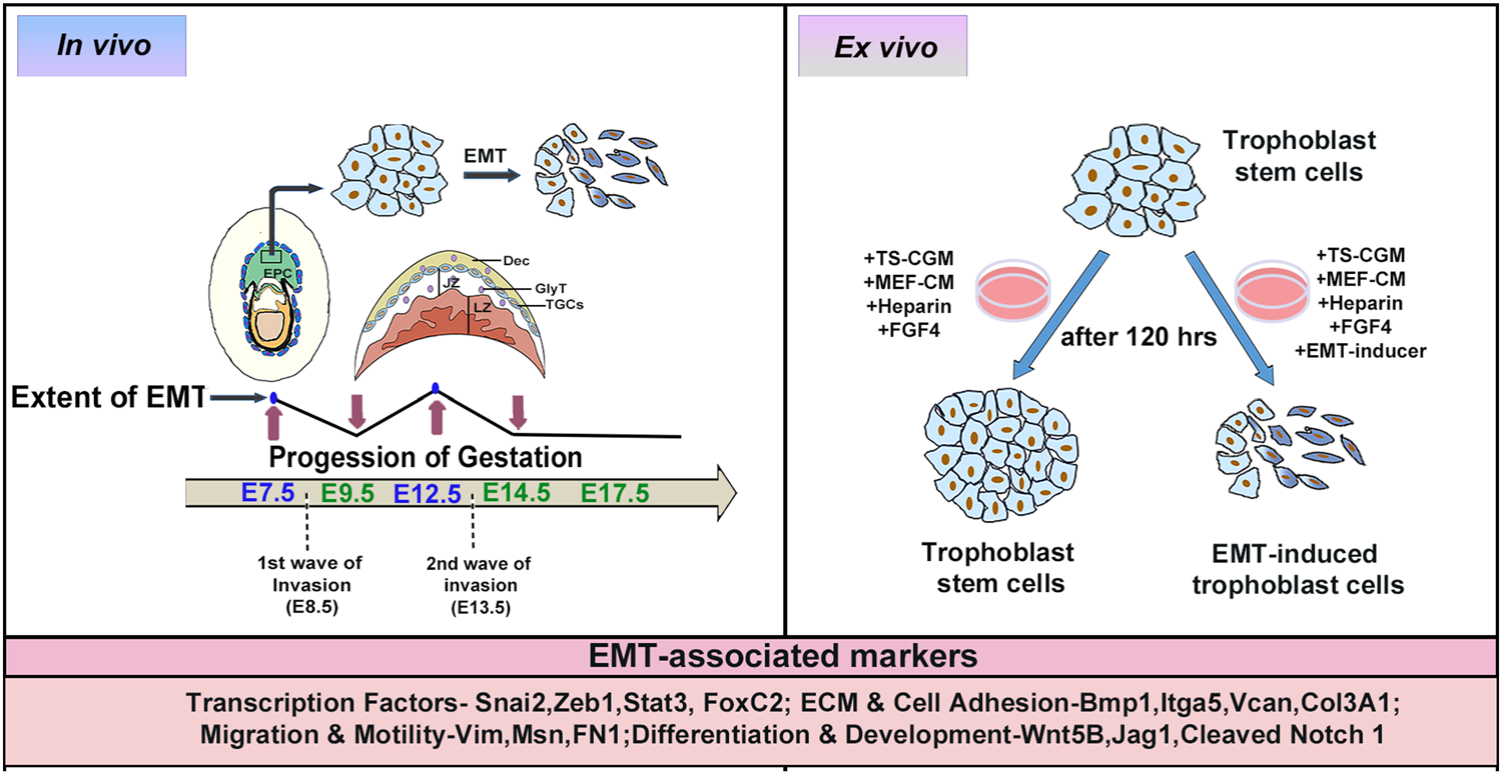
Schematic representation summarizing EMT during placental development and TS cell differentiation. Two EMT waves, one on E 7.5 and another on E12.5, are distinct during placentation in mice in vivo. It is imperative that these EMT waves precede waves of trophoblast invasion during placental morphogenesis. Molecular signatures associated with induction of EMT in placental trophoblast is recapitulated ex vivo in trophoblast stem cells during EMT induction. *Dec* decidua, *JZ* junctional zone of the placenta, *LZ* labyrinth zone of the placenta, *GlyT* glycogen trophoblast cells, *TGC* trophoblast giant cells, *CGM* complete growth medium, *MEF-CM* mouse embryonic fibroblast conditioned media, *ECM* extracellular matrix.

In conclusion, two EMT waves, one on E 7.5 and another on E12.5, were evident during placentation in mice in vivo. It seems likely that these EMT waves precede waves of trophoblast invasion during placental morphogenesis. Molecular determinants associated with induction of EMT in placental trophoblast is recapitulated ex vivo in trophoblast stem cells during EMT induction (Fig. 8).

## Discussion

Trophoblast invasion is an integral part of placental morphogenesis and development. It has been elegantly demonstrated using first trimester human placental-derived CBT and EVT that EMT-marker genes are upregulated in EVT^22^. Furthermore, invasiveness in human EVT was shown to be associated with elevated mesenchymal markers^24–26^. Following this trail of information, in the current study we analysed the status quo of EMT in placental development throughout gestation in mice and also validated these results using ex vivo TS cell model system.

Our data showing heightened expression of the transcription factors, SNAI2, ZEB1, STAT3 and FOXC2 on E7.5 and their remarkable decline on E9.5 indicates occurrence of enhanced EMT on E7.5. The first wave of trophoblast invasion into the decidua associated with formation of definitive placenta initiates on E7.5. Therefore, our data and existing literature^6,27^ indicate that along with rapid cell division and differentiation of trophoblast progenitor cells, TS cells of EPC undergo EMT, which is required for placental morphogenesis. Promoter analysis of these transcription factors showed that there are putative binding sites of SNAI2, ZEB1 and STAT3 on *Bmp1*, *Itgα5*, *Vcan*, *Col3A1*, *Vim*,* Msn*, *Fn1*, *Wnt5B and Jag1* gene promoter, whereas, FOXC2 binding sites were found in all of the above gene promoters except *Msn*. This in silico finding is in line with our experimental results where expression of these genes consistently coincided with the expression of these transcription factors.

Our report is based on analysis of 84 EMT-associated genes present in the PCR-array. These genes were chosen mostly from literature on EMT in cancer cells. For example, overexpression of Snai2 in pancreatic ductal adenocarcinoma induces EMT by suppressing epithelial markers and up-regulating mesenchymal markers as a result of which there is increase in migration capacity^28^. Knockout of Zeb1 led to loss of colonizing capacity, decreased invasiveness, ability of metastasis in pancreatic cancer^29^. Stat3 and NF-κB activation is responsible for EMT-induction and inactivation of both decreases the expression of Snail and Zeb-2 leading to inhibition of EMT in Gall bladder cancer^30^. Similarly, Increased FOXC2 has been reported to be related with cadherin switch and high VIM expression in patients with hepatocellular carcinoma^31^. There might be other genes, related to trophoblast-EMT that were not analysed using this profile. However, consistent evidence of similar gene groups using array, real time PCR and western blotting across multiple biological samples strongly supports trophoblast-EMT during placental development.

Our findings on EMT gene expression from E12.5 to E17.5 demonstrated that EMT associated gene expression gradually decreases after E12.5. In mice, the second wave of trophoblast invasion initiates at mid-gestation. These data indicate that EMT gene expression in placenta is primarily associated with trophoblast invasion. Furthermore, our data also confirmed EMT gene expression throughout gestation showing that EMT is an ongoing process during placental morphogenesis. These data are indicative of dynamic mechanism of trophoblast invasion, which is a hallmark of haemochorial placentation. Nonetheless, further experimentation is required to identify the trophoblast cell population that undergo EMT during placentation.

ECM and cell adhesion markers are essential in maintaining cell structural integrity. Alteration in these proteins disrupt the physical and biochemical properties of ECM during EMT eventually leading to change in cellular morphology^32^. Knock down of BMP1 has been reported to decrease the invasiveness, cell migration capacity of clear cell renal cell carcinoma in vivo and in vitro^33^. Overexpression of ITGA5 induces EMT and thereby increase the expression of mesenchymal marker in oral squamous carcinoma^34^. Similarly, Vcan is also associated with EMT in gastric cancer cells. Downregulation of VCAN inhibits cell migration and expression of VIM, which is an important marker of mesenchymal cells^35^. According to TCGA dataset, EMT markers have been reported to be strongly related with collagen genes in glioma and COL3A1 knockdown inhibits EMT, cell migration, invasion in glioma cell^36^. Our data on increased expression of ECM and cell adhesion markers in early gestation E7.5 and during mid-gestation E12.5, as well as in EMT-induced trophoblast cells are in line with above findings confirming trophoblast-EMT during placentation.

Migration and Motility associated markers, Vim, is a crucial biomarker of EMT^37,38^. Increased expression of Msn were reported to be responsible for remodelling of actin filament and cell morphological changes during EMT^39^. Similarly, Fn1 is also involved in cellular migration^40^. Heightened expression of Vim, Msn and Fn1 on E7.5 and E12.5, therefore, indicate migration of cells during placental development. Immunohistochemical localization of vimentin on E7.5 IS shows presence of marker near the EPC (ectoplacental cone) suggesting first wave of trophoblast invasion and on E12.5 placental tissues vimentin expression is maximum at decidual layer of placenta which suggests the second wave of trophoblast invasion in which cells from junctional zone migrates and invades decidua for spiral artery remodelling. WNT5B upregulation was shown upon TGFβ-mediated EMT-induction via Wnt-signaling and during cataract development^41^. Similarly, overexpression of JAG1 in colorectal cancer has been reported to induce EMT and promote metastasis^42^. Notch1 was shown to be up-regulated in the patients with chronic kidney disease characterized by low expression of e-cadherin and high expression of collagen-1 associated with EMT induction^43^. Our data showing high expression of these differentiation and development markers on E7.5, E12.5 provides the proof of the principle that EMT induction is associated with trophoblast invasion during placentation.

Our ex vivo data on the ability of TS cells to undergo EMT-like phenotypic (morphological) changes in presence of appropriate stimulus is a second layer of evidence that trophoblast cells undergo EMT during placental development. This is further affirmed by similar changes in the genotypic markers of EMT as observed in placenta. Taken together, data represented in this report highlight that EMT is an ongoing process throughout placental life during gestation. Initiation of two distinct waves trophoblast invasion have been demonstrated previously on E8.5^44^ and on E13.5^8^. Our data on heightened EMT marker gene expression on E7.5 and E12.5 indicate that EMT precedes trophoblast invasion during placentation. Morphological as well as genotypic changes leading to EMT-signature gene expression in TS cells upon treatment with EMT inducer cocktail provides a further line of evidence regarding occurrence of EMT in placental trophoblast cells during its development. These results have broad biological implications in understanding placental development, in particular, under pathological conditions leading to disruption of pregnancy.

## Materials and methods

### Animal tissue collection

Mouse placentae were collected from timed pregnant mice. Female Swiss albino mice were caged overnight with stud male in 3:1 ratio. E0.5 of pregnancy was confirmed by the presence of copulatory plug in the vagina of the female mice. Implantation sites without the uterus were collected on E7.5 and E9.5 and placental tissues were dissected out from female pregnant mice on different days of pregnancy (, E12.5, E14.5, E17.5). Tissue samples were snap frozen and kept in – 80 °C for RNA and Protein isolation. Indian Institute of Chemical Biology Animal Ethics and Care Committee approved all procedures for handling and experimentation with rodents as per guidelines set forward by the Committee for the Purpose of Control and Supervision of Experiments on Animals (CPCSEA), Govt. of India (http://cpcsea.nic.in).

### Cell culture

Mouse blastocyst-derived TS cells were a kind gift from Professor Janet Rossant, Hospital for sick Children, Toronto, Canada. TS cells were cultured as described before^27,45^ using 30% TS basal medium, containing RPMI-1640 (Sigma Aldrich, USA) supplemented with 20% FBS, 1% Penicillin–Streptomycin,1% Glutamax (Invitrogen, USA), 1 mM sodium pyruvate, 100 μM β-mercaptoethanol (Sigma Aldrich, USA), and 70% mouse embryonic fibroblast (MEF) conditioned medium. TS media was further supplemented with 25 ng/ml FGF4 (R&D Systems, USA), and 1 mg/ml heparin (Sigma Aldrich, USA).

EMT was induced in TS cells by supplementing TS media with StemXVivo EMT inducing media supplement (100×) (R&D Systems, USA). Cells were seeded at 20,000 cells per 35 mm dish and were maintained either in stemness condition or in EMT-inducing condition for 120 h, with media changed in every 48 h in presence of 5% CO_2_ at 37 °C in a humidified incubator.

### Quantitative RT^2^ profiler PCR array

A large-scale quantitative real-time PCR array was performed using a mouse EMT RT^2^ Profiler™ PCR Array (catalogue No. PAMM-090ZA, SABiosciences-Qiagen, Germany) as per the manufacturer’s instructions as described before^46^. The PCR array included 84 SYBR Green-optimized primers specific to EMT-associated genes that were assessed in a 96-well format. RNA was isolated from mouse IS (E7.5 and E9.5) using TRIzol reagent and were purified using a RNeasy mini kit (Qiagen, Germany). The concentration and quality of the RNA was measured using a Nano-Drop 2000 Spectrophotometer (Thermo Fischer Scientific, USA). Genomic DNA elimination was followed by the cDNA synthesis using RT^2^ first strand kit (Qiagen, Germany) and real-time array was done using RT^2^ SYBR Green q-PCR Master Mix (Qiagen, Germany). Samples were normalized using the house-keeping genes, which showed no change between samples.

### RNA isolation and quantitative real-time PCR

Total RNA was isolated from tissue samples and cells using TRIzol reagent (Invitrogen, USA) as per manufacturer’s protocol. Concentration and quality of extracted RNA was measured using Nano-drop 2000 Spectrophotometer and cDNA was prepared using M-MLV Reverse Transcription kit (Invitrogen, USA). Quantitative real-time PCR reaction was done using tenfold dilution of cDNA and Power SYBR Green PCR Master Mix (Applied Biosystems, USA). Reactions were run in a 7500 real-time PCR system (Applied Biosystems, USA) using standard PCR condition described previously^47^. Primers used for real-time PCR are enlisted in Table 1. *Gapdh* was used as a reference gene for normalization of the gene of interest. Relative expression of RNA was calculated using 2^–∆∆Ct^ method as described before^48^. Three biological replicates were used for each experiment.

### Protein isolation and western blot

Mouse placental tissue samples (E7.5, E9.5, E12.5, E14.5, E17.5) were homogenized using tissue homogenizer in RIPA buffer containing (20 mM-Tris–HCl, pH7.5, 150 mM NaCl, 1 mM Na_2_EDTA, 1 mM EGTA, 1% NP40, 1% sodium deoxycholate, 2.5 mM sodium Pyrophosphate, 1 mM β-glycerophosphate, 0.2 mM PMSF, and 1 mM sodium orthovanadate) supplemented with protease inhibitor cocktail (Sigma-Aldrich, USA). Protein extraction from TS cells was done by trypsinzing the cell with 0.05% trypsin in order to isolate pure stem cell population followed by washing of cell pellet using Dulbecco’s phosphate-buffered saline (DPBS) (Thermo Fisher Scientific, USA). Cells were then lysed in RIPA buffer. For isolating protein from EMT-induced trophoblast cells, RIPA buffer was directly added to the cells, incubated for 15 min and were scraped off. Cell lysates were then sonicated for 30 s per pulse, three pulses per sample at 10 MHz with 1 min gap between each pulse. Samples were then centrifuged at (14,000 rcf for 10 min at 4 °C) and supernatant was collected. Concentration of each protein sample was estimated using Bio-Rad Protein Assay Reagent (Bio-Rad, USA). the membrane was blocked, incubated with primary and secondary antibodies, respectively using standard protocol. Sixty microgram of total protein were fractioned by 10% sodium dodecyl sulfate–polyacrylamide gel electrophoresis (SDS-PAGE) and then transferred onto a PVDF membrane (Millipore, USA). Post-transfer the membrane was cut according to the relevant molecular weights of different markers, following which the membrane was blocked, incubated with primary and secondary antibodies, respectively using standard protocol. Antibodies used for western blot analysis are enlisted in Table 3. Chemiluminescence signal detection was done using an ECL reagent, Luminate Forte (Millipore, USA). Images were acquired with the Biospectrum 810 imaging system (UVP LLC, Upland, CA, USA). Normalization was done using GAPDH (loading control) and NIH Image J Software was used for densitometry analysis of the samples. Three biological replicates were used for each experiment.

**Table 3:** List of antibodies used in western blot analysis.

Sl. no.	Primary antibody	Source	Cat. no.	Dilution
1	SNAI2	CST	9585T	1:1000
2	ZEB1	Bethyl Lab	A301-922A-T	1:1000
3	STAT3	CST	4904	1:2000
4	FOXC2	SCBT	sc-515472	1:250
5	BMP1	ABCAM	ab205394	1:500
6	ITGA5	CST	4705	1:1000
7	VCAN	ABCAM	ab19345	1:2000
8	COL3A1	SCBT	sc-514601	1:200
9	VIM	CST	5741s	1:1000
10	MSN	CST	3150S	1:1000
11	FN1	SCBT	sc-6952	1:250
12	WNT5B	ABCAM	ab93134	2.5:1000
13	JAG1	CST	2620T	1:1000
14	NOTCH1	CST	4147	1:1000
15	GAPDH	CST	14C10	1:4000

Sl. no.	Secondary antibody	Source	Cat. no.	Dilution
1	Goat anti-rabbit IgG HRP conjugated	Bethyl Lab	A120-101P	1:10,000
2	Donkey anti-goat IgG HRP conjugated	Bethyl Lab	A50-101P	1:10,000
3	Goat anti-mouse IgG HRP conjugated	Bethyl Lab	A90-116P	1:10,000
4	Anti-rabbit IgG HRP-linked	CST	7074S	1:2000

### Hoechst and phalloidin staining

Assessment of change in cytoskeleton and nuclear size were done using Hoechst–Phalloidin staining as described before^49^. Cells were seeded a coverslip kept in 35 mm cell culture dish and cultured for 120 h, media was changed every 48 h. After 120 h cells were fixed using 4% paraformaldehyde (Sigma-Aldrich, USA) for 15 min, washed with PBS 3 times and stained with Phalloidin (DyLight™ 554, CST) in methanol at working concentrations (1:200) for 20 min in dark. Cells were then washed with PBS 3 times for 5 min each followed by incubation with Hoechst (2 µg/ml) for 15 min in dark. Cells were washed with PBS for 6 times 5 min each. Imaging was done at 200× magnification using a Leica DMi8 epifluorescence microscope.

### Immunofluorescence

Immunofluorescence was performed as described previously^6^. Briefly, 5000 TS cells were seeded on a coverslip in 35 mm cell culture dish. Cells were cultured in stemness condition or EMT-inducing condition 120 h with media change in every 48 h. The media was discarded and cells were washed with PBS once. Cells were fixed with 4% paraformaldehyde for 15 min at room temperature followed by washing with PBS three times for 5 min each. Cells were blocked using blocking buffer (1 × PBS, 5% serum, 0.3% Triton X-100) for one hour. Cells were then incubated with antibody dilution buffer (1 × PBS, 1%BSA, 0.3% Triton X-100) containing vimentin antibody (1:100) for 1.5 h at room temperature. Cells were then washed with 1 × PBS (3 times) for 5 min each. Subsequently, cells were incubated with TRITC-conjugated anti-rabbit (1:100) secondary antibodies in antibody dilution buffer for 1.5 h at room temperature in dark. Cells were washed with 1 × PBS (3 times) for 5 min, then nuclei were stained using Hoechst (2 µg/ml) for 15 min in dark. Cells were then washed with 1 × PBS 6 times for 5 min each. Imaging was done at 200× magnification using a Leica DMi8 epifluorescence microscope.

### Immunohistochemistry

Immunohistochemical analysis was performed on 10 µm cryosections of E7.5 implantation site and E12.5 placental tissue using the Vectastain Elite ABC HRP kit (Vector Laboratories, Inc., Burlingame, California). Briefly, cryo-sections were fixed using ice-cold acetone solution for 5 min. Then the sections were washed under tap water for 5 min and endogenous peroxidases were quenched off using 0.3% hydrogen peroxide. Samples were washed with PBS for three times and blocked with goat serum for 45 min. Tissue sections were then incubated with primary antibody (VIMENTIN/ZEB1) at 1:100 dilutions for 1.5 h and negative control were incubated without primary antibody for 1 h. Post-incubation, sections were washed with PBS three times and again incubated in a biotinylated anti-rabbit secondary antibody (1:200) for 30 min, followed by incubation with avidin–biotin-peroxidase complex Elite ABC reagent for 30 min. Then tissue sections were washed with PBS three times and the signal was developed by incubating the sections using peroxidase AEC substrate kit (Vector Laboratories, Inc.). Mayer’s Hematoxylin was used for counterstaining and images were captured using Leica DMi8 epifluorescence microscope.

### Statistical analysis

Data are represented as mean ± SEM from at least three biological replicates. A Non-parametric Mann–Whitney U test was used to compare between groups. p < 0.05 is considered to be statistically significant for all experiments. Statistical evaluations were done using Graph pad prism (Version 8.0) software. Asterisks in the figures indicate significance levels: *p < 0.05, **p < 0.01, and ***p < 0.005.

### Ethics approval

Indian Institute of Chemical Biology Animal Ethics and Care Committee approved all procedures for handling and experimentation with rodents as per guidelines set forward by the Committee for the Purpose of Control and Supervision of Experiments on Animals (CPCSEA), Govt. of India (http://cpcsea.nic.in). In addition, ARRIVE guidelines were followed when conducting and reporting the study.

## Supplementary Information

Supplementary Information 1.

Supplementary Information 2.

## Data Availability

All data presented in the study are included in the article and in the Supplementary Material.
