# Supplementary material for: Molecular determinants of epithelial mesenchymal transition in mouse placenta and trophoblast stem cell
Source: Sci Rep. 2023 Jul 6;13:10978. doi: 10.1038/s41598-023-37977-2 (PMC10325982; doi:10.1038/s41598-023-37977-2)

Figure S1: Original full length blots are shown along with the corresponding cropped blots

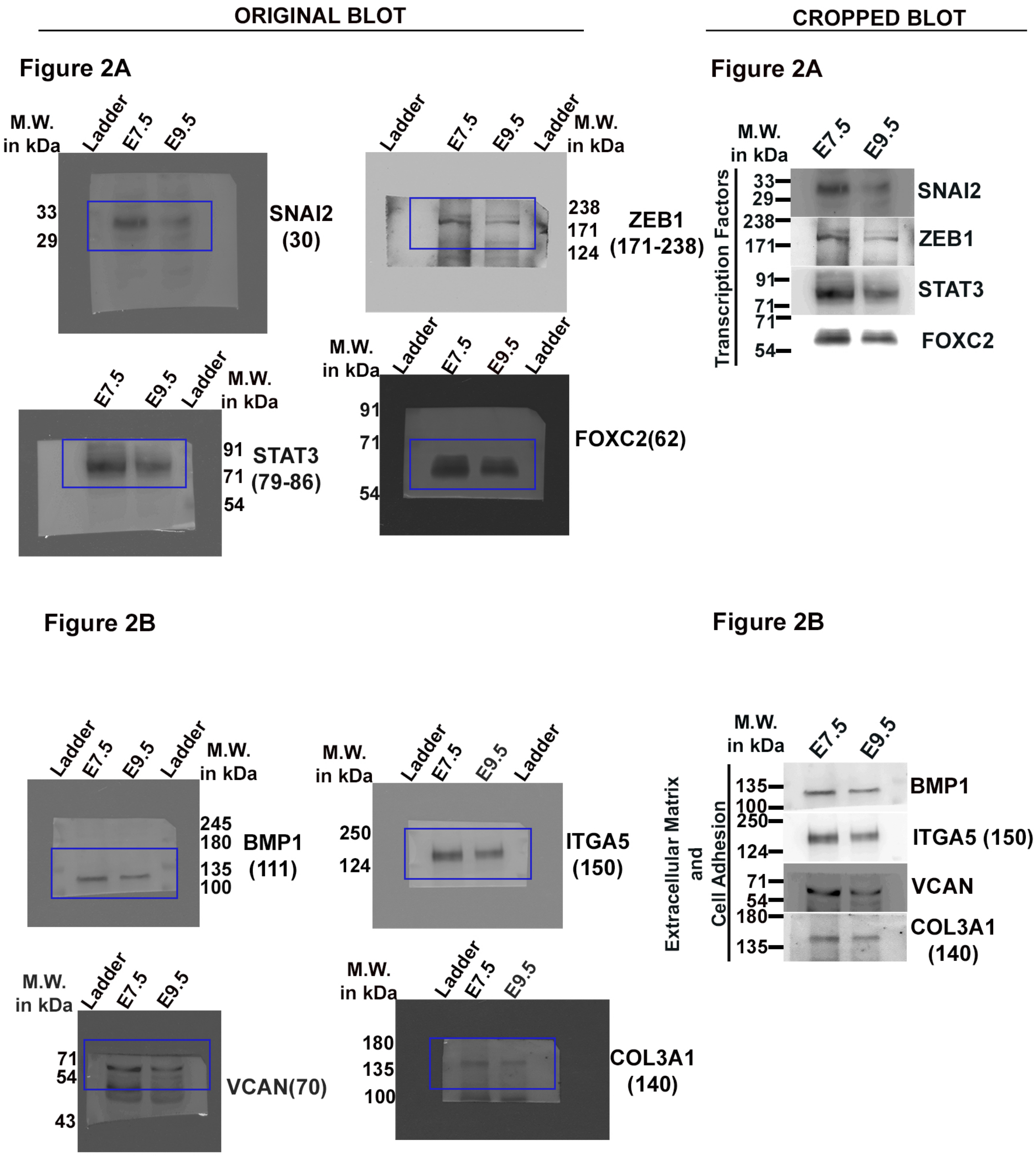

ORIGINAL BLOT

CROPPED BLOT

Figure 2C

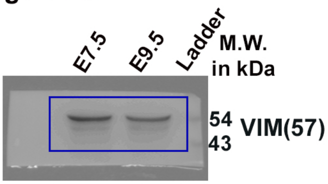

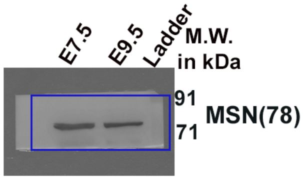

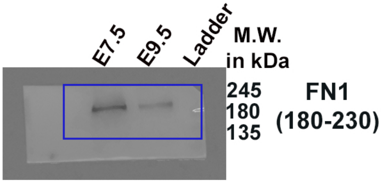

Figure 2C

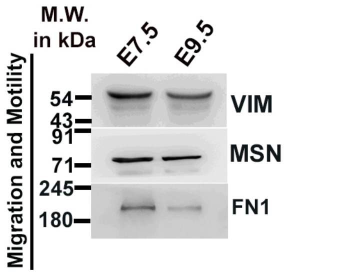

Figure 2D

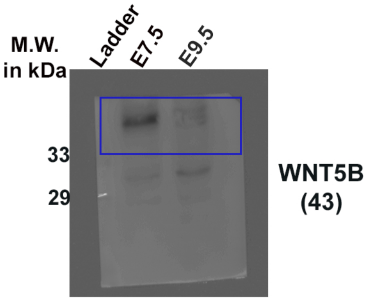

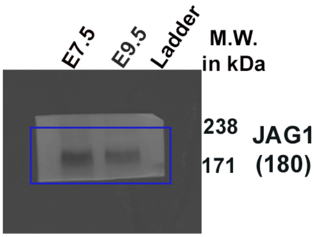

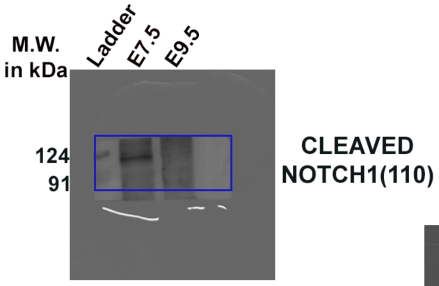

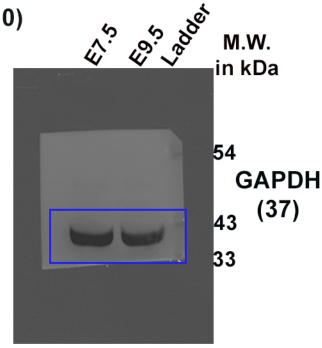

Figure 2D

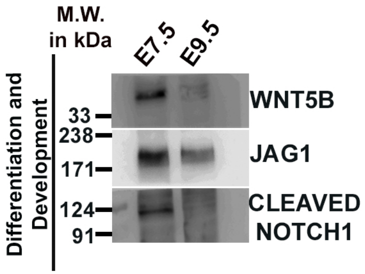

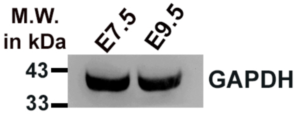

ORIGINAL BLOT

CROPPED BLOT

Figure 3A

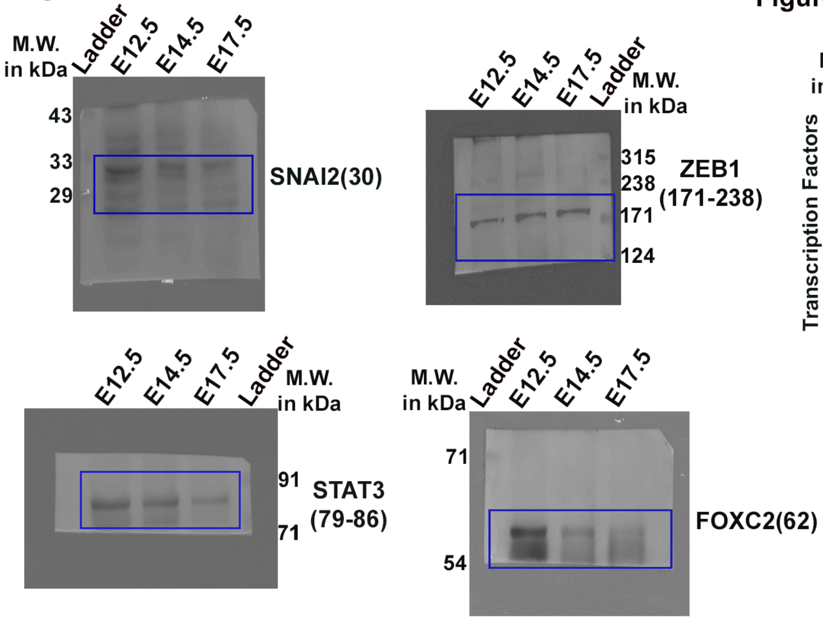

Figure 3A

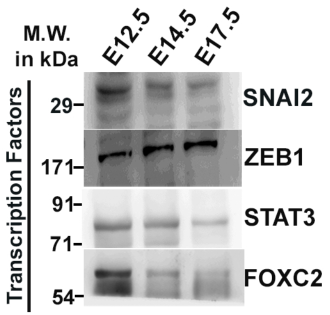

Figure 3B

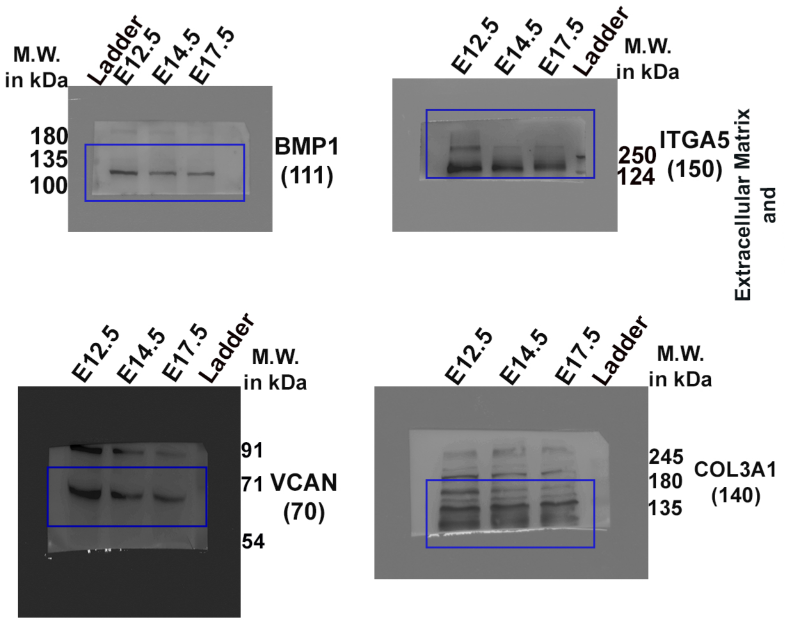

Figure 3B

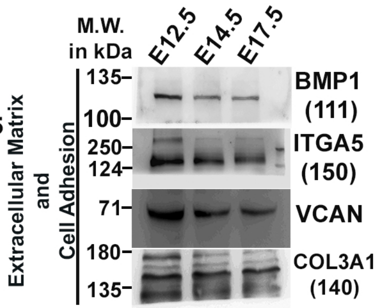

ORIGINAL BLOT

CROPPED BLOT

Figure 3C

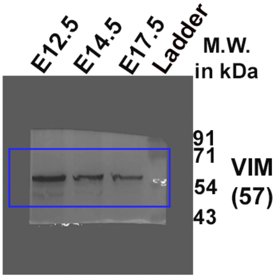

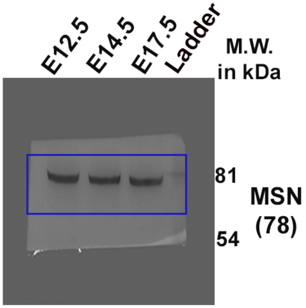

Figure 3C

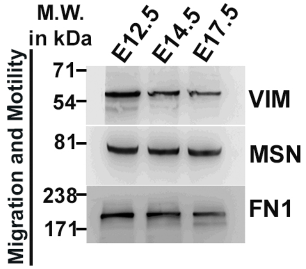

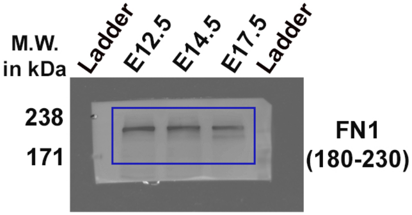

Figure 3D

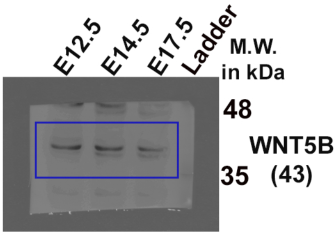

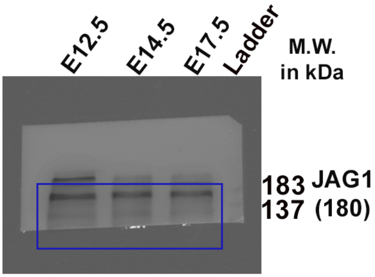

Figure 3D

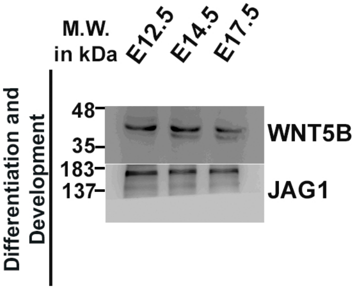

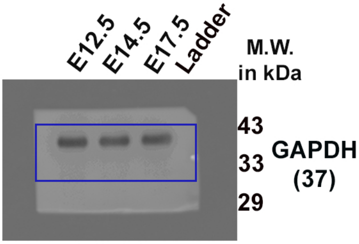

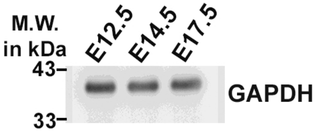

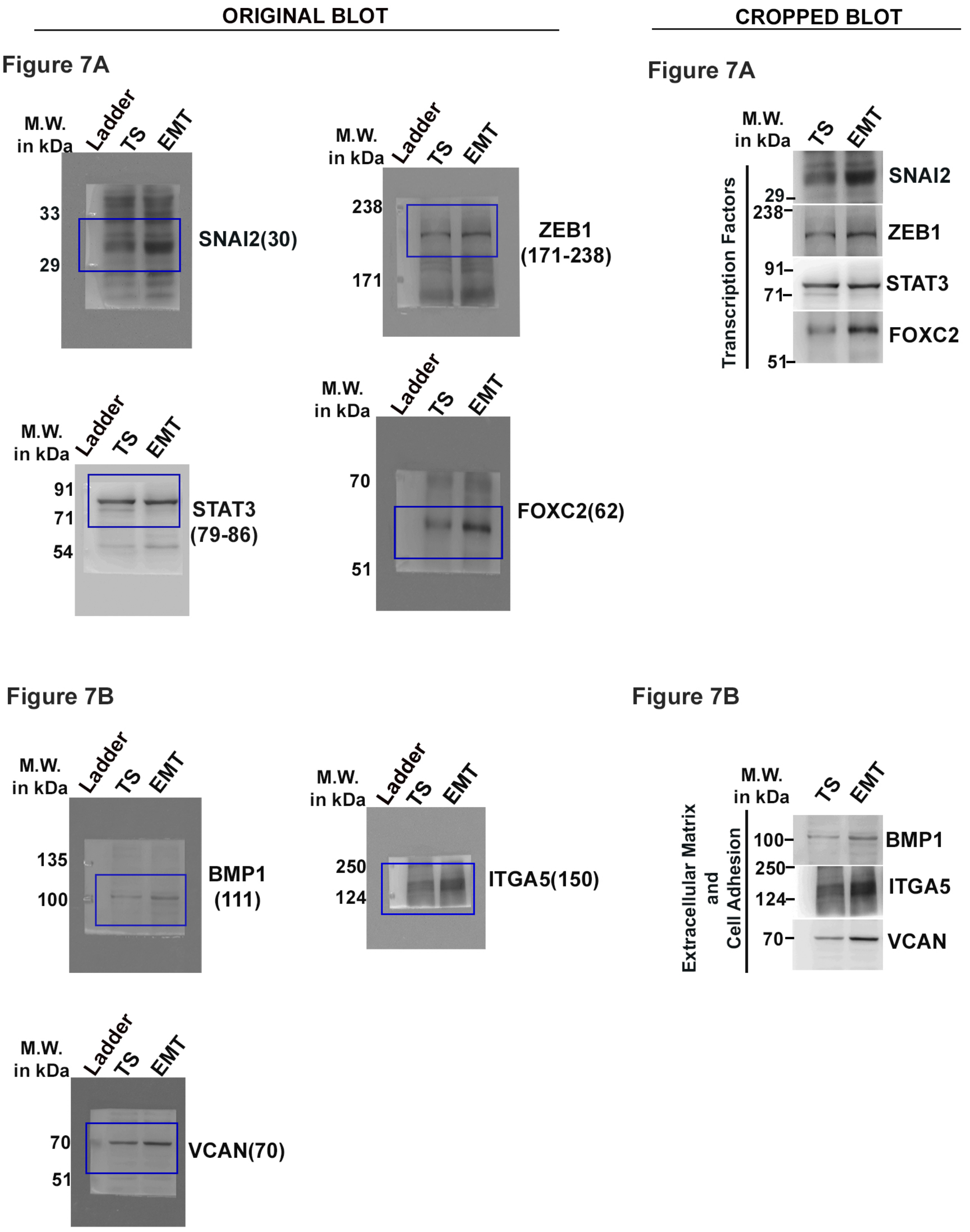
ORIGINAL BLOT
CROPPED BLOT
Figure 7A
M.W. in kDa
Ladder
TS
EMT
33
29
SNAI2(30)
238
171
ZEB1 (171-238)
91
71
54
STAT3 (79-86)
70
51
FOXC2(62)
Figure 7A
Transcription Factors
SNAI2
ZEB1
STAT3
FOXC2
Figure 7B
135
100
BMP1 (111)
250
124
ITGA5(150)
70
51
VCAN(70)
Figure 7B
Extracellular Matrix and Cell Adhesion
BMP1
ITGA5
VCAN

ORIGINAL BLOT

CROPPED BLOT

Figure 7C

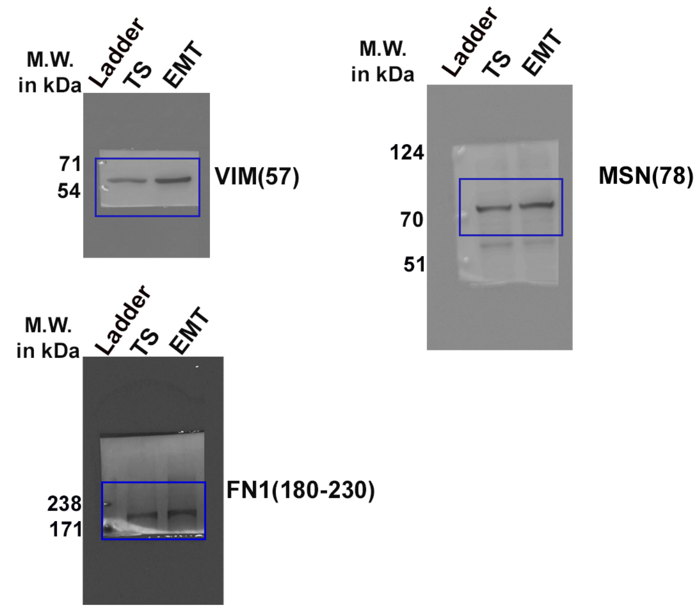

Figure 7C

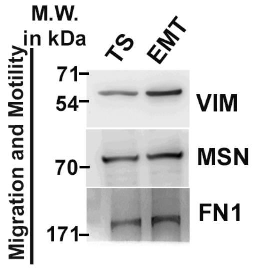

Figure 7D

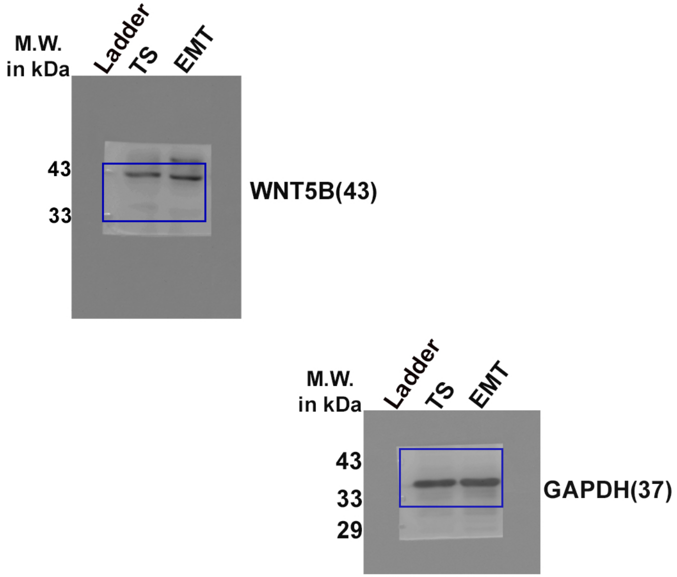

Figure 7D

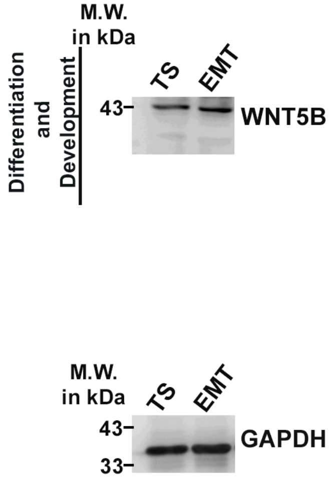

Supplement: Supplementary file 1 — Supplementary Information 1. [file 41598_2023_37977_MOESM1_ESM.pdf]
