# Supplementary material for: Molecular determinants of epithelial mesenchymal transition in mouse placenta and trophoblast stem cell
Source: Sci Rep. 2023 Jul 6;13:10978. doi: 10.1038/s41598-023-37977-2 (PMC10325982; doi:10.1038/s41598-023-37977-2)
Supplement: Supplementary file 2 — Supplementary Information 2. [file 41598_2023_37977_MOESM2_ESM.docx]

**Table S1: List of down-regulated genes in E9.5**

| Sl.No. | Gene Symbol | Accession No. | Mean Ct E7.5 | Mean Ct  E9.5 | Fold Change |
| --- | --- | --- | --- | --- | --- |
| 1 | Zeb1 | NM_011546 | 21.9 | 34.32 | -1260.99 |
| 2 | Wnt5a | NM_009524 | 17.44 | 23.25 | -12.928 |
| 3 | Vcan | NM_001081249 | 18.72 | 24.36 | -11.3703 |
| 4 | Wnt5b | NM_009525 | 22.82 | 28.4 | -11.0047 |
| 5 | Mmp2 | NM_008610 | 17.94 | 23.2 | -8.7937 |
| 6 | Col3a1 | NM_009930 | 18.55 | 23.55 | -7.6942 |
| 7 | Rgs2 | NM_009061 | 14.97 | 19.97 | -7.3311 |
| 8 | Esr1 | NM_007956 | 23.46 | 28.36 | -6.8372 |
| 9 | Snai2 | NM_011415 | 18.31 | 23.17 | -6.682 |
| 10 | Plek2 | NM_013738 | 22.19 | 27.04 | -6.6515 |
| 11 | Timp1 | NM_011593 | 18.56 | 23.3 | -6.1278 |
| 12 | Col1a2 | NM_007743 | 15.4 | 20.11 | -6.0163 |
| 13 | Notch1 | NM_008714 | 22.62 | 27.22 | -5.5737 |
| 14 | Zeb2 | NM_015753 | 17.03 | 21.56 | -5.2924 |
| 15 | Rac1 | NM_009007 | 14.59 | 18.76 | -4.1401 |
| 16 | Ilk | NM_010562 | 16.01 | 20.08 | -3.8559 |
| 17 | Camk2n1 | NM_025451 | 21.31 | 25.37 | -3.8078 |
| 18 | Vim | NM_011701 | 15.38 | 19.43 | -3.7944 |
| 19 | Sparc | NM_009242 | 12.85 | 16.86 | -3.6987 |
| 20 | Jag1 | NM_013822 | 20.82 | 24.82 | -3.69 |
| 21 | Ptp4a1 | NM_011200 | 18.58 | 22.57 | -3.647 |
| 22 | Stat3 | NM_011486 | 18.45 | 22.38 | -3.4751 |
| 23 | Ptk2 | NM_007982 | 19 | 22.9 | -3.432 |
| 24 | Fzd7 | NM_008057 | 21.88 | 25.76 | -3.3881 |
| 25 | Nudt13 | NM_026341 | 21.73 | 25.54 | -3.2151 |
| 26 | Pdgfrb | NM_008809 | 21.49 | 25.29 | -3.1919 |
| 27 | Itga5 | NM_010577 | 16.14 | 19.93 | -3.1648 |
| 28 | Msn | NM_010833 | 16.72 | 20.5 | -3.1606 |
| 29 | Ctnnb1 | NM_007614 | 15.91 | 19.68 | -3.1409 |
| 30 | Mst1r | NM_009074 | 21.2 | 24.98 | -3.1369 |
| 31 | Fn1 | NM_010233 | 16.35 | 20.01 | -2.9009 |
| 32 | Gsk3b | NM_019827 | 18.5 | 22.13 | -2.8404 |
| 33 | Col5a2 | NM_007737 | 19.11 | 22.61 | -2.6732 |
| 34 | Itgb1 | NM_010578 | 15.79 | 19.27 | -2.5499 |
| 35 | Desi1 | NM_134095 | 17.91 | 21.35 | -2.4968 |
| 36 | Mitf | NM_008601 | 17.75 | 20.95 | -2.1099 |
| 37 | Bmp1 | NM_009755 | 20.01 | 23.01 | -2.0256 |
| 38 | Tgfb1 | NM_011577 | 19.47 | 22.6 | -2.0240 |
| 39 | Cdh2 | NM_007664 | 26.89 | 29.88 | -2.0187 |
| 40 | Mmp9 | NM_013599 | 18.68 | 21.4 | -2.0120 |
| 41 | Foxc2 | NM_013519 | 26.17 | 28.72 | -2.07 |
